# Parents’ concerns and beliefs about temperature measurement in children: a qualitative study

**DOI:** 10.1186/s12875-020-01355-y

**Published:** 2021-01-07

**Authors:** Elizabeth Morris, Margaret Glogowska, Fatene Abakar Ismail, George Edwards, Susannah Fleming, Kay Wang, Jan Y. Verbakel, Ann Van den Bruel, Gail Hayward

**Affiliations:** 1 Nuffield Department of Primary Care Health Sciences, University of Oxford, Radcliffe Observatory Quarter, Woodstock Road, Oxford, UK; 2 Institute of Cardiovascular and Medical Sciences, University of Glasgow, 18 Alexandra Parade, Glasgow, G31 2ER UK; 3 Academic Centre for Primary Care, Department of Public Health and Primary Care, KU Leuven, Kapucijnenvoer 33 J, 3000 Leuven, Belgium

**Keywords:** Fever, Child, Thermometers, Primary health care, Qualitative research

## Abstract

**Background:**

Nearly 40% of parents with children aged 6 to 17 months consult a healthcare professional when their child has a high temperature. Clinical guidelines recommend temperature measurement in these children, but little is known about parents’ experiences of and beliefs about temperature measurement. This study aimed to explore parents’ concerns and beliefs about temperature measurement in children.

**Methods:**

Semi-structured qualitative interviews were conducted from May 2017 to June 2018 with 21 parents of children aged 4 months to 5.5 years, who were purposively sampled from the METRIC study (a method comparison study comparing non-contact infrared thermometers to axillary and tympanic thermometers in acutely ill children). Data analysis followed a thematic approach.

**Results:**

Parents described the importance of being able to detect fever, in particular high fevers, and how this then influenced their actions. The concept of “accuracy” was valued by parents but the aspects of performance which were felt to reflect accuracy varied. Parents used numerical values of temperature in four main ways: determining precision of the thermometer on repeat measures, detecting a “bad” fever, as an indication to administer antipyretics, or monitoring response to treatment. Family and social networks, the internet, and medical professionals and resources, were all key sources of advice for parents regarding fever, and guiding thermometer choice.

**Conclusions:**

Temperature measurement in children has diagnostic value but can either empower, or cause anxiety and practical challenges for parents. This represents an opportunity for both improved communication between parents and healthcare professionals, and technological development, to support parents to manage febrile illness with greater confidence in the home.

**Supplementary Information:**

The online version contains supplementary material available at 10.1186/s12875-020-01355-y.

## Background

“High temperatures” are one of the most commonly reported symptoms in children under 5 [1]. Nearly 40% of parents with children aged 6 to 17 months consult a healthcare professional when their child has a high temperature [1]. International guidelines commonly recommend temperature measurement in these children [2, 3], but little is known about parents’ experiences of and beliefs about temperature measurement.

Previous research on childhood fever has focussed on parents’ approach to management [4–6], and experiences of healthcare consultations for this issue [7]. Episodes of fever are concerning for parents [8], seeing a healthcare professional may provide reassurance [7], and more reliable advice on self-management strategies is required [7, 9]. This is echoed in research with primary care physicians, who feel management of childhood fever represents a considerable workload, and report disparity between levels of parental concern and their own impression of illness severity [10].

However, no studies to date have focussed solely on the detection of childhood fever, nor explored the role of technology in temperature measurement. While the presence of fever is a cause for parental concern [8], it is important to understand how temperature measurement itself is seen by parents, why they check their child’s temperature, and how they interpret the results. Additionally, with the increasing availability of self-monitoring technologies at home, as well as the expanding remote methods for interacting with healthcare providers (which have become more common during recent unprecedented clinical demands and infection control restrictions [11]), understanding the role of temperature measurement technology in both self-management and interactions with healthcare professionals is crucial.

To address this gap in the literature, this study employed a qualitative methodology to explore parents’ views and experiences of temperature measurement in children, using semi-structured interviews.

## Methods

The methods used for this study have been described previously [12, 13] and are summarised below.

### Aims, design, recruitment and sampling

The METRIC study [12], a thermometer method comparison study, recruited 401 acutely unwell children from general practice consultations in Oxfordshire, UK, comparing two different non-contact infrared thermometers (NCITs) with axillary and tympanic thermometers in children aged ≤5 years with acute illness [12]. This nested qualitative study aimed to explore parents’ feelings about different types of thermometers and their use, as well as their opinions and experiences of temperature measurement in children, and its perceived role, through individual semi-structured interviews. Research team members (EM and FAI) purposively sampled parents of children enrolled in the METRIC study who gave consent to contact, to achieve maximum variation in gender, age of parent, age of child, ethnicity, number of children in the household, and whether their child had had a history of fever during the episode of presentation to healthcare when they had been recruited to the METRIC study. This last category was felt to be important as it could impact the immediacy of their reflections on temperature measurement and experience with thermometers, and expectations from healthcare professionals in this context. These participants were contacted by telephone to re-discuss their participation in the qualitative study, and arrange a time that would suit them to conduct the interview by phone. Researchers continued recruitment until the core research team agreed data saturation had been achieved, with no new issues emerging and sufficient explanation for the categories generated being reached.

### Data collection

Researchers conducted semi-structured interviews, following a flexible topic guide developed by the research team and Patient and Public Involvement (PPI) panel (Additional file 1). The topic guide was informed by the aims of the study, the existing literature (primarily parents’ experiences of childhood fever [4, 5, 7, 8, 14]), and the expertise of the multidisciplinary research team. The topic guide evolved in response to emerging themes and ongoing PPI involvement.

Interviews were conducted by telephone (20) or face-to-face (1), from May 2017 to June 2018. All participants gave recorded verbal informed consent.

Two researchers trained in qualitative methodology conducted the interviews; EM (a female clinical researcher and salaried General Practitioner (GP)) and FAI (a female research assistant). Consistency and rigour was ensured by regular discussion between researchers, discussion with the PPI panel, and review of transcripts and topic guides by the study team (EM, FAI, GH, MG).

Interviews lasted an average of 10–15 min duration. Interviews were audio-recorded and transcribed verbatim by an external transcription company and checked for any inconsistencies against the original recording by the research team.

The study was approved by the South Central – Berkshire Research Ethics Committee (Ref: 17/SC/0068).

### Data analysis

Data were analysed thematically, following Braun and Clarke’s approach [15], with the assistance of NVivo 11 Qualitative Data Analysis software for data management. The researchers read and re-read the transcripts to familiarise themselves with the data, noting initial thematic areas, then conducted open coding. Inductive reasoning was used to draw out and identify categories within the data, which were then refined and developed into themes. Codes and themes were checked by two researchers (EM, MG) and interpreted in discussion with the wider research team.

## Results

65.1% of parent participants in the METRIC study consented to be re-contacted for inclusion in the qualitative study. Of those re-contacted, only two declined to participate at this stage, citing childcare needs (1) and time since initial study participation being more than 1 week (1). Demographics of the participants are summarised in Table 1; Table 2 details individual participant characteristics. Parents’ views of the individual thermometers, and ideal device attributes, have been reported previously [12, 13]; the findings presented here relate only to parents’ concerns and beliefs about temperature measurement in children.

**Table 1:** Participant Demographics

Parental characteristics		N
**Gender**	Male	6
	Female	15
**Age**	20–30	3
	31–40	11
	> 40	3
**White British Ethnicity**	Yes	15
	No	6
**Case characteristics**		
**Age of child**	≤12 months	9
	12-24 months	7
	> 24 months to 5 years 6 months	5
**History of fever this episode**	Yes	5
	No	16
**Total no. of children in household**	1	12
	2	9

**Table 2:** Individual participant characteristics

Interview ID	Parental role	Age bracket (years)	Number of children	White British?	History of fever this episode
**i1**	Father	–	2	Yes	No
**i2**	Mother	20–30	1	Yes	Yes
**i3**	Father	–	1	Yes	No
**i4**	Mother	31–40	2	Yes	No
**i5**	Mother	20–30	2	Yes	No
**i6**	Mother	31–40	1	Yes	No
**i7**	Mother	31–40	2	Yes	No
**i8**	Mother	31–40	2	Yes	No
**i9**	Mother	–	1	No	No
**i10**	Father	–	1	Yes	Yes
**i11**	Mother	31–40	1	Yes	No
**i12**	Mother	31–40	2	No	Yes
**i13**	Mother	> 40	1	No	No
**i14**	Father	31–40	2	No	Yes
**i15**	Mother	31–40	1	Yes	No
**i16**	Mother	31–40	2	No	No
**i17**	Mother	31–40	1	Yes	No
**i18**	Mother	31–40	1	Yes	No
**i19**	Mother	> 40	1	Yes	No
**i20**	Father	20–30	1	No	Yes
**i21**	Father	> 40	1	Yes	No

Table 3 outlines the key themes.

**Table 3:** Main themes

Themes	Subthemes	
The importance of temperature measurement to parents	Diagnosis of (high) fever Indication for action Situation-specific value and cost	
Parents’ use and interpretation of results	Accuracy of readings Interpretation of numerical values	Definition of accuracy Anxiety about usage Indication for action Monitoring response to treatment Thresholds for a “bad” / high fever
Sources of advice	Family and social networks Books and the internet Medical professionals and resources	Advice about fever Advice about thermometers and temperature measurement
Home use patterns and parental experience	Parental factors influencing use of thermometers Motivation for healthcare consultations	

### The importance of temperature measurement to parents

#### Diagnosis of (high) fever

Parents described diagnosing “fever” as important, with its presence or absence causing concern or reassurance respectively. Some parents explicitly linked the presence/absence of a fever in a child to diagnostic value for clinicians:

*“They take the temperature..to know if it was something viral or a bacteria” (i9).*

As well as identifying illness as “febrile” or “not febrile”, parents agreed that temperature measurement could identify a “high” fever, which was accepted as signifying a “bad” or more serious illness, and therefore important to recognise.

*“I know as a first time mum if they feel a bit hot you think...I need to get the thermometer out and check the temperature…because obviously a very high temperature can be very dangerous so the earlier you catch it the better”. (i2).*

#### Indication for action

Temperature measurement was also important to parents as an indicator for action – such as giving antipyretics or seeking medical review.

*“I think going over 40 was a key one. So 39.08 I would probably try and manage that at home, hitting over 40 I would be going to the emergency room” (i12).*

For some parents, measuring their child’s temperature and detecting a high value appeared to either drive, or validate their need for medical contact to provide reassurance.

*“The temperature had been a little over 40.. Ibuprofen brought it down to 37 or 38..but we still decided to make an appointment at the doctor just to make sure that it wasn’t anything serious..I suppose what I was expecting from the doctor was probably confirmation it wasn’t anything serious and reassurance that what we were doing was okay” (i10).*

#### Situation-specific value and cost

Many parents interviewed had home thermometers, from forehead thermometer strips, axillary and tympanic to NCITs. Some parents described purchasing a thermometer with the arrival of a new baby, often prompted by family or friends. However, many were triggered to purchase a thermometer during an episode of acute illness, with a situation-specific change in how important they felt accurate temperature measurement was. Several parents equated the cost of the devices to their perceived reliability, seeking the reassurance of a more expensive device for situations where they were more concerned about their child, and being happier with cheaper options if they did not feel their child was ill.

*“If my children were both well I would probably.. just take the £10 one, but then when your child is very sick.. I think that I would be more likely to be willing to part with a lot more money in..an emergency situation and will go out specifically to buy one” (i12).*

### Parents’ use and interpretation of the results

Parents’ use and interpretation of the results focussed on interpreting the accuracy of the readings, and the numerical values themselves.

#### Accuracy of readings

##### Parental definition of “accuracy”

A key emergent theme was the importance to parents of thermometer accuracy. The definition of what they would consider “accuracy” varied widely. For some, the essential feature was the ability to detect presence or absence of a fever; other parents looked for reproducibility of the readings on repeat testing:

*“If I was to take a reading in one ear and then in the other ear..if that is showing quite a consistent reading...I think I would feel like it was working correctly” (i7).*

However, few had pre-formed ideas about how similar the numbers themselves would have to be to be “accurate”; some had a rule of thumb they used, but many were less clear.

*“if they’re more than a degree apart then I start wondering and then I’ll do it again” (i10).*

*“they weren’t too far off each other so I was happy with the results” (i4).*

##### Anxiety about usage

Parents expressed concerns regarding whether how they used the thermometer might affect its accuracy:

*“I’m never sure if I’ve put it far enough into her ear or not far enough in…I’m not confident that I wouldn’t miss something by not being able to use it perfectly” (i17).*

A common anxiety was that their lack of training or imperfect usage would lead to inaccurate readings, missing a fever or illness. This motivated some parents to seek a medical review, for the reassurance of a healthcare professional confirming the temperature measurement, and highlighting parent’s perception of the crucial role of temperature measurement in assessing their child’s illness.

*“I have had some times when..I don’t even think it’s working correctly or I don’t think it’s that accurate so it is always best to stop to get it checked by the doctor because they can obviously do it correctly” (i12).*

#### Interpretation of numerical values

Numerical values of temperature were used in three further ways by parents.

##### Indication for action

Some parents described using numerical thresholds as an indication for treatment or action,

*“I think going over 40 was a key one. So yes 39.08 I would probably try and manage that at home, hitting over 40 I would be going to the emergency room” (i12).*

where others used measures like “height” of temperature or how quickly it had evolved:

*“It’s more seeing the difference…it’s seeing how long it’s taken her to get up to that temperature and that sort of thing” (i18).*

#### Monitoring response to treatment

Most parents described repeating measurements to monitor response to treatment with antipyretics, looking for a reduction in temperature, rather than achieving a particular target.

*“if the temperature responds to Paracetamol or Ibuprofen..if it comes down within half an hour or so then generally there’s not too much to worry about but if it doesn’t respond to either of those two then it could be something more serious” (i10).*

#### Thresholds for a “bad” fever

Parents who cited a numerical value commonly used 38, 39 or 40 °C (reported by three, four, and five participants respectively) as their threshold for concern or a “bad” fever. However, many (especially first-time parents) reported the challenge of recalling what a “normal” temperature was:

“*In actual fact I can’t even remember it from one week to the next, so basically every time they have a fever I have to Google what is actually acceptable and what is not” (i20).*

Some parents found removal of the numbers altogether, and interpretation of the results by one of the NCITs (Thermofocus 800), more helpful:

*“So the blue one had a smiley face…just kind of symbols rather than numbers…I much preferred that because even though I would count myself as a fairly knowledgeable person, when she’s unwell I’m a bit stressed I can’t then remember what counts as a temperature” (i6).*

Despite the importance placed on the accuracy of temperature measurement, many parents still described relying more on their child’s appearance to tell them whether their child had a “bad” fever:

*“I think I go with a general look of how they look to me and how poorly they look and how they behave. And then I touch them and if they’re really, really [hot]…knowing immediately if it is really high fever” (i16).*

### Sources of advice

Parents reported seeking advice about thermometers, and fever when their children were unwell, from three main sources: family and social networks, the internet, and medical professionals or United Kingdom National Health Service (UK NHS) resources.

#### Advice about fever

If their child had a fever, many parents first turned to friends or relatives. This was reported predominately by first-time parents, who found accessing other experiences of parenting reassuring.

*“I would call my mum. Normally my mum would be the one who will say I really think you ought to take her to the doctors if it’s something I’m not sure about. Yeah she’s had three children, she’s also got six grandchildren so I feel that..she’s got experience behind her.” (i15).*

Many of the parents would also consult the internet for advice. For some this was an automatic first port of call, describing the reassurance of using an endorsed, reliable site like the NHS website. Others described the internet as something to use only if they were not seriously concerned, or to help them make decisions about when to seek medical help.

*“Probably initially I would probably consult Dr Google but if I was really concerned I would call the doctors surgery.” (i15).*

The most commonly reported source of advice for acute febrile illness was contact with the medical profession. Around half of parents would call a first point of contact such as NHS 111 to seek advice, whereas others, especially first-time parents, would want to contact their doctor for reassurance.

*“If she was to get a fever I would ring 111 or the doctors to seek advice. And then normally they recommend to give a bit of Calpol to try and help bring it down but my first thought would be to ring one of them to see if I could get advice from them first.” (i18).*

There was also an awareness of telephone based resources as a source of advice, which could both help them and avoid using valuable NHS resources.

*“Well if, if it’s something I can do for her myself..and they can tell me what to do I find that very helpful. Because then obviously I’m not just taking her down to the doctors to waste their time as well as my own when it can be something that’s easily done at home.” (i18).*

For others, a face-to-face appointment with their GP would be a secondary step, only if their child was not improving, or they had a higher level of concern.

While some parents were concerned that their child might need something other than over-the counter treatments or supportive care, other parents were seeking reassurance by seeing the doctor – not necessarily a concrete diagnosis or prescription.

*“I wouldn’t really care too much about the doctor knowing what was wrong just wanting them to reassure us it’s nothing major.” (i9).*

#### Advice about thermometers

When seeking advice about which thermometer to use at home, parents varied between familiarity with what experienced family members used (mainly tympanic thermometers), and a more consumer-based approach. Parents were primarily looking for internet-based resources, although did not always find these available or easy to access:

*“I looked into this because I wanted to get the right thermometer..I ended up reading a lot of stuff on the Braun website and then I tried finding information on Which Online..I normally like to try and find some independent body that’s actually got information..people who have got a vested interest in marketing something seem to be the ones who are providing the most information. So I’d like the NHS to have this sort of information accessible.” (i9).*

Parents would have appreciated endorsement of one device as the “best” one to use. Endorsement was identified both implicitly, by identifying the models used in healthcare settings, and explicitly, by seeking recommendations from recognised sources.

*“When [he] was first born I went out and bought a relatively cheap one..and the readings were just rubbish..it was obvious he had a temperature and it wasn’t detecting that..I went out and I bought the one that they use in the doctors surgeries and I’m confident that that is accurate.” (i8).*

### Home use patterns and parental experience

#### Parental factors influencing use of thermometers

Several parents identified that their use of thermometers and temperature checks at home varied with their child’s age, and that having a thermometer available at home increased their confidence and frequency of use.

*“I think if you have something at home you will have the tendency to check more often especially when they’re little, I know as a first time mum if you they feel a bit hot you think…I need to get the thermometer out and check the temperature..” (i2).*

They also described the importance of practicality of the technology, especially for new parents:

*“I mean I’m not so worried now but when you’re a new parent you just want to know you’re not just giving them medicine for the sake…I just really wanted to know did they have a fever or not and I think you’re always maybe, not in a panic but you’re not necessarily relaxed so a thermometer that you can use that basically does it quickly without you having to think too much about it” (i20).*

#### Motivation for healthcare consultations

Parents described how their response to temperatures and temperature measurement had changed over time. In particular, both the age of their child and experience with second children changed their likelihood of seeking a medical review:

*“I think you just gain more confidence the more children you have and the older they get.. whereas if you’d asked me that question when he was 11/12 months… I would say as soon as I detect a high temperature I’m at the doctors regardless of what else is happening.” (i8).*

They also mentioned how temperature measuring devices could impact on healthcare-seeking behaviour, through features like inbuilt guidance on severity of fever (colour coding the numerical results), and parental faith in the devices through medical endorsement.

*“It’s good to have a RAG status [Red Amber Green] on the thermometer.. but that’s quite reassuring because obviously amber is up to, I’ve got a feeling it’s 38 something and then anything I think above that is red and again that just sort of makes you feel a bit more reassured let’s try the Calpol route let’s see how his general behaviour is before we sort of jump the gun and start calling especially at weekends, out of hours” (i8).*

## Discussion

### Summary of main findings

In this study we found that parents valued being able to diagnose fever through temperature measurement, particularly high fevers, which were regarded as a marker of more serious illness. They routinely include temperature measurement when assessing if their child is unwell, as they have seen in consultations with a healthcare professional. Parents attach significance both to the absolute temperature values recorded, and their change over time and with treatment, as well as inferring from the readings device accuracy and competency of their own usage. Parents reported that their frequency of usage and interpretation of temperatures had changed as their children got older, and with second or subsequent children. They expressed a wish for advice and medical endorsement regarding both which thermometer to use, and how to interpret the findings, which would then influence when and how they sought healthcare professionals’ input.

### Strengths and limitations

To our knowledge this is the first study to specifically explore parents’ beliefs and concerns regarding temperature measurement in children. Telephone interviews facilitated contact with parents who might not otherwise have been able to participate due to practical challenges and childcare commitments. All parents had been part of the METRIC study and so had recent concrete experiences of illness in their child and temperature measurement on which they could reflect in the interview.

As with all research of this nature, interviews were restricted to those who agreed to participate, which may have excluded parents of children who were more seriously unwell at initial presentation, or selected those with more interest in temperature measurement in children. All participants were from Oxfordshire, and there was limited ethnic diversity (reflecting the demographics of this region), but we were able to gain a range of participants on other dimensions (Table 1), such as parental age, and number of children.

### Comparison with other literature

Our findings reflect the trend in more recent literature [4, 16] of parents now engaging with monitoring and assessment of their children, using technology such as thermometers, alongside their assessment of behaviour (previously more heavily relied upon [17]), to detect fever and illness. This may in part be due to the availability of more affordable thermometers, or the implicit endorsement of seeing temperature measurement form part of a clinical assessment. Additionally, with the increase in telephone triage in primary care, parents are asked whether they have objectively measured their child’s temperature, [18, 19] which may suggest to parents that this is something they should do.

Previous studies have found marked variability in parents’ numerical definitions of a “fever” [14], with one study reporting that 63.1% of parents identified temperatures at which they defined fever that were either below or above the recognised definition of a fever (38 degrees) [16]. Confusion regarding the threshold for fevers may be partly due to the variability in national guidance; both UK National Institute for Health and Care Excellence (NICE), and World Health Organisation (WHO) guidelines use thresholds of both 38 and 39 degrees in their recommendations regarding febrile illness in infants [2, 20], while the American Academy of Pediatrics highlights different thresholds for “normal” temperatures with different methods of thermometry (e.g. rectal, ≤38 °C, versus oral, ≤37.2 °C) [21]. In our study, while parents’ definitions, and personal thresholds for concern, still showed significant variation, there was more marked focus on the concerning features of a “high” fever and responsiveness of the fever to time, or medication. This may reflect a greater awareness of “serious infections”, with the possible contribution of campaigns to increase sepsis awareness in the general public, alongside a growing evidence base supporting the role of temperature measurement in identification of severe infections [22].

A qualitative study of primary care physicians’ experiences of childhood fever in out-of-hours care found that doctors perceived inability of parents to employ self-management strategies to increase the number of consultations [7]. However, our data suggest that parents were generally well informed about self-management strategies, and keen to attempt to use these at home. Yet many parents still sought healthcare contact, primarily seeking reassurance of their diagnosis of a fever, that it was not a sign of serious illness, or that they were managing the illness correctly, as has previously been described [8, 14].

However, our data also show that some parents felt a clinical review was necessary because of their doubts about their own measurement methods. Parents seeking reassurance that they had taken the temperature correctly identified a need for more guidance in this area, which could reduce their healthcare contacts. This corroborates the findings of a systematic audit of information in thermometer leaflets, highlighting the lack of consistent, evidence based information for parents about fever detection and management [9].

While we found that consultation with professionals and their own social circles remain key sources of advice for parents, the first points of contact are changing. In 2007, parents reported that “the internet was not generally a source of child health information”, with books being more commonly accessed [5]. This contrasts with the internet resources, online social networks, and telephone advice lines reported here. This suggests that any new resources for parents should be designed to be accessed across these platforms.

## Conclusions

This study highlights an unmet need for medical endorsement regarding the best temperature measurement devices and improved guidance on their use at home. As highlighted in this study, for many significant purchases relating to children or home, there are independent review sites which provide comparisons of the existing technology, but no such resources currently exist for thermometers. Previous studies of parents’ management of childhood illnesses identified a context-dependent interest in medical issues, seeking information only when their child was ill [23]. While some parents in this study described seeking information on the best thermometer to buy when preparing for the arrival of a new baby, many purchased a thermometer during an episode of acute illness, and demonstrated clear situation-specific value around the costs and reliability of a thermometer, according to how unwell they felt their child was.

The findings from this study highlight that home temperature measurement technology for children has the potential to significantly impact healthcare contacts in this context. Recent devices enable visual reinforcement of fever thresholds through colour coding, feedback on how the device should be placed or used, and potentially more precise measurements, which all provide reassurance and support to parents that they are assessing and managing their child’s illness appropriately. Such technology provides the opportunity to support and empower parents further with the self-management strategies they are already embracing – and may also improve early detection (and access to healthcare) for those with serious illness.

## Supplementary Information

**Additional file 1.**

## Data Availability

The datasets generated and analysed during the current study are not publicly available to avoid any potential for participants to be identified, but are available from the corresponding author on reasonable request.

## Abbreviations

GP: General Practitioner (primary care physician)
METRIC: “Measuring Temperature In Children” Study
NCIT: Non-Contact Infrared Thermometer
NHS: National Health Service
NICE: National Institute of Health and Care Excellence
PPI: Patient and Public Involvement
WHO: World Health Organisation
