# Supplementary material for: Parents’ concerns and beliefs about temperature measurement in children: a qualitative study
Source: BMC Fam Pract. 2021 Jan 7;22:9. doi: 10.1186/s12875-020-01355-y (PMC7791980; doi:10.1186/s12875-020-01355-y)
Supplement: Supplementary file 1 — Additional file 1. [file 12875_2020_1355_MOESM1_ESM.docx]

**Additional file 1 – Topic Guide Summary**

**Parent/guardian interviews: Topic Guide**

**The questions and prompts below are detailed for example purposes. Prompts were only used if necessary to help the participant to elaborate. Where possible, the open questions were used throughout.**

**This paper presents only the findings related to 'Parents concerns and beliefs about temperature measurement in children'; other data has been presented elsewhere ((12, 15 - see manuscript references).**

*1. Introduction*

Explain purpose of interview and audio recording procedures.

Answer any questions. Begin audio recording, with consent.

Gain informed consent from participant.

*2. Introductory questions:*

- Could you tell me about the time leading up to your appointment, and what made you bring *XXX(child’s name)* to the GP?
- What were you hoping for when you came to the GP or OOH GP?

(Did you expect your child would have a temperature measurement? Did you want your child to have a temperature measurement?)

*3. First thoughts about the thermometers*

- How do you think your child felt about having the temperature measurement with the non-contact thermometer no. 1 and no. 2; with the *under arm* thermometer and with the ear thermometer?
- Was there anything that put you off any of the temperature measurements?
- *When you are at the GP surgery, if your child cries when they are being examined (for example, when the GP checks their temperature), how do you feel about this?*

*4. Future use of the thermometers*

- Which thermometer do you think should be used in the future?
  (In what situations? *Eg at the GP surgery/healthcare setting? Or at home?) How about for yourself, what makes you decide which sort of thermometer should be used?*
- Would you consider using a non-contact thermometer in the home environment or suggesting they use one at a childcare facility? What features make you more or less likely to do this?
- *Would the cost affect whether you think GPs should use it? Is it important to you what measurement device the GP uses?*

*5. Wider explorative questions*

- What are your thoughts, or feelings, about measuring temperature in children more generally?
  (Do you think these measurements are offered frequently enough? Do you want your child to have more or less temperature measurements?)
- What makes you suspect that your child might have a fever?
- What makes you decide to measure your child’s temperature?
- When you have measured their temperature, does this affect how you feel?
- *When you think they have, or have measured, their temperature, what is it that will make you decide to book an appointment with the GP?*
- Once you have measured their temperature, how often will you re-check it? What do you think affects your decision to re-check the temperature? (Does this depend on whether you have given them medication at all? Would the timing of this affect how you decide when to re-check their temperature?)
- What is it that would make you say your child has a “bad” fever/temperature?
- Where do you go for advice about your child if they have a temperature/fever?
- Do you tend to give your child medicine if they have a fever? If so, which? Do you follow any advice in particular about giving your child medicines if they have a fever?
- Some people have been explaining to us that the accuracy of a thermometer is important to them.. when you check your child’s temperature at home, do you trust the measurement? What methods do you use to measure their temperature?
- What does an “accurate” reading mean to you? What would it take to reassure you about the accuracy of any type of thermometer?
- Do you think having a thermometer you trusted at home would change how you felt when your child had a fever, or change what you might do?

5. Any other remarks?

*6. Close*

Thank participant.

End audio recording.
